# Novel Genetic Variants Associated with Diabetic Neuropathy Risk in Type 2 Diabetes: A Whole-Exome Sequencing Approach

**DOI:** 10.3390/ijms26136239

**Published:** 2025-06-28

**Authors:** Noémi Hajdú, Dóra Zsuzsanna Tordai, Ramóna Rácz, Zsófia Ludvig, Ildikó Istenes, Magdolna Békeffy, Orsolya Erzsébet Vági, Anna Erzsébet Körei, Bálint Tóbiás, Anett Illés, Henriett Pikó, János P. Kósa, Kristóf Árvai, Péter András Lakatos, Péter Kempler, Zsuzsanna Putz

**Affiliations:** 1 Department of Internal Medicine and Oncology, Semmelweis University, 1083 Budapest, Hungary; tordai.dora@semmelweis.hu (D.Z.T.); racz.ramona@semmelweis.hu (R.R.); zsofiludvig@gmail.com (Z.L.); istildi78@gmail.com (I.I.); bekeffy.magdi95@gmail.com (M.B.); vagiorsi@gmail.com (O.E.V.); korei.anna@semmelweis.hu (A.E.K.); balint.tobias@gmail.com (B.T.); illes.anett@semmelweis.hu (A.I.); kosa.janos@semmelweis.hu (J.P.K.); kristof.arvai@gmail.com (K.Á.); lakatos.peter@semmelweis.hu (P.A.L.); kempler.peter@semmelweis.hu (P.K.); putz.zsuzsanna@semmelweis.hu (Z.P.); 2 Hungarian Research Network SE-ENDOMOLPAT Research Group, 1085 Budapest, Hungary

**Keywords:** type 2 diabetes, neuropathy risk, genetic variants

## Abstract

The pathogenesis of diabetic neuropathy involves complex interactions between metabolic and genetic factors. This study aimed to identify novel genetic variants associated with neuropathy risk in type 2 diabetes through reanalysis of whole-exome sequencing data. We identified seven new SNPs with significant associations, including intronic variants in *TTN*, *PLCB1*, *CCNI*, and *CDC34* and a 5′-upstream variant in *BTG2*. These variants are implicated in muscle elasticity, neurotransmission, endothelial regeneration, and apoptosis resistance, suggesting multifaceted genetic contributions to neuropathy development. These findings enhance our understanding of diabetic neuropathy and may support future advances in risk stratification and therapy development.

## 1. Introduction

Microvascular complications—such as nephropathy, retinopathy, and neuropathy—significantly contribute to the increased mortality among patients with diabetes [1]. Diabetic neuropathy, in particular, carries a poor prognosis and affects up to 50% of patients [2]. Its clinical characteristics are extremely varied, and as a result, a multidisciplinary approach is necessary for patient care [3].

Distal symmetric polyneuropathy (DSPN) is a common chronic microvascular complication of both type 1 and type 2 diabetes [4]. DSPN severely impairs quality of life by increasing the risk of lower-limb ulcers, amputations, and falls [5]. The resulting complications impose a substantial economic burden on healthcare systems [6]. According to the meta-analysis by Vági et al., overall mortality nearly doubles among diabetic patients with distal symmetric polyneuropathy compared to those without [4].

Cardiovascular involvement is one of the most clinically relevant aspects of autonomic neuropathy. It causes a wide range of symptoms and complaints, such as postural hypotension, reduced exercise tolerance, intraoperative cardiovascular instability, and silent myocardial ischemia or infarction [7,8]. It is well known that the presence of cardiovascular autonomic neuropathy (CAN) significantly increases cardiovascular mortality [9]. According to the retrospective cohort study by Vági et al., the presence of cardiovascular autonomic neuropathy is associated with a significant increase in the relative risk of mortality [10].

Timely recognition and appropriate comprehensive treatment are critically important in the care of diabetic patients, so patients should always be educated about the risks of chronic complications of diabetes and the importance of screening [11]. Optimal metabolic control is fundamental in the prevention of diabetic neuropathies [11]. The UKPDS study, conducted with newly diagnosed type 2 diabetic patients, demonstrated that the presence of chronic complications is closely associated with glycemic control. A 1% reduction in HbA1c is associated with decreased risks of microvascular and macrovascular complications, cardiovascular disease, and overall mortality [12].

However, findings from the ACCORD study indicate that intensive glycemic control only has a limited effect on the prevention of microvascular complications [1]. This raises the possibility that other factors, including genetic susceptibility, also play a role in the development of such complications.

In a previous study, our research group performed whole-exome sequencing on type 2 diabetic patients with and without neuropathy [13]. We successfully identified five genetic variants that may influence the risk of neuropathy in patients with type 2 diabetes: the rs2032930 and rs2032931 variants in the RecQ-mediated genome instability protein 2 (*RMI2*) gene and the rs604349 variant in the myosin-binding protein H-like (*MYBPHL*) gene were found to increase the risk of neuropathy by 22–49-fold, while the rs917778 SNP in the multivesicular body subunit 12B (*MVB12B*) gene and the rs2234753 SNP in the retinoic acid X receptor alpha (*RXRA*) gene were found to reduce the probability of neuropathy to 0.07–0.08 [13].

The aim of our current study is to further analyze the whole-exome data in silico to identify additional genetic variants that may influence the appearance of neuropathy.

## 2. Results

We compared data from patients with type 2 diabetes and neuropathy to those of patients with type 2 diabetes but without neuropathy.

The characteristics of the two study groups are provided in Table 1. The average age was higher in patients with type 2 diabetes and neuropathy compared to those without neuropathy. In contrast, no significant differences were found between the groups regarding the duration of diabetes, body mass index (BMI), HbA1c levels, or lipid profile.

The results of whole-exome sequencing are presented in Table 2 [14].

We discovered seven additional genetic variants that are significantly linked to an increased risk of developing diabetic neuropathy. The rs922984, rs2291313, and rs4471922 variants in the titin gene; the rs6086563 SNP in the phospholipase C-beta 1 gene; the rs4241602 variant in the cyclin I gene; and the rs2396295, variant along with rs892204, in the cell division cycle 34 gene were found to be associated with a 22- to 26-fold increase in risk. Additionally, one other variant, the rs6682221 variant of the anti-proliferation factor 2 (*BTG2*) gene, appeared to have a protective effect, reducing the risk of neuropathy to approximately 0.045. Table 2 summarizes the chromosomal locations and positions of these genetic variants, along with their corresponding odds ratios (ORs), which indicate the probability of developing neuropathy.

## 3. Discussion

We successfully identified additional genetic variants that may alter the risk of developing diabetic neuropathy.

### 3.1. rs922984, rs2291313, and rs4471922 SNPs of Titin Gene

rs922984 is a missense variant, while rs2291313 and rs4471922 are intronic SNPs within the titin gene. Titin is a structural protein that supports myofibrillar assembly during myogenesis, determines the passive elasticity of the muscle, and carries out various signaling functions. It interacts with 170 different protein ligands, including telethonin, α-actinin, sAnk1, filamin C, nebulin, tropomyosin, αB-crystallin, FHL1, FHL2, calpains 1 and 3, and muscle ankyrin repeat proteins (MARPs). Through these interactions, it influences key cellular processes, such as phosphorylation, calcium binding, and myosin binding [15]. Titin maintains sarcomere structure both longitudinally and radially and contributes to the active contraction of the striated muscle [16]. Titin, as a giant sarcomeric protein, also plays a significant role in the diastolic function of the heart [17].

Titin has two main isoforms: N2B and N2BA. The ratio of these isoforms is primarily regulated by an alternative splicing factor, RNA-binding motif protein 20 (RBM20). RBM20 is a muscle-specific splicing factor and serves as a key regulator of titin splicing in sarcomeric muscles, including cardiac and skeletal muscle [18].

The composition of the titin isoform determines passive myocardial stiffness. Increased titin N2B isoform expression may impair the contractile function of the heart and lead to diastolic dysfunction. [19]. In animal studies, *RBM20* knockout mice and rats expressed only the largest titin isoform, N2BA-G. Furthermore, loss-of-function mutations in this splicing factor led to increased expression of the N2BA isoform, and mice carrying *RBM20* mutations developed idiopathic dilated cardiomyopathy [18,20]. In healthy adults, the smaller titin isoform, N2B, is predominantly expressed in the cardiac muscle under the regulation of the RBM20 splicing factor. However, in the absence of RBM20, only the larger N2BA isoform is expressed. These findings indicate that RBM20 is a key dose-dependent regulator of titin splicing in the heart. In a study by Chaoqun Zhu et al., it was demonstrated that RBM20 expression is correlated with insulin levels, thereby influencing titin splicing. Insulin can stimulate protein synthesis through the mammalian target of rapamycin (mTOR) pathway, which increases RBM20 expression, while inhibition of phosphoinositide 3-kinase (PI3K) or mTOR decreases RBM20 levels [17].

Krüger et al. [21] reported an increased proportion of the stiffer N2B titin isoform in cardiomyocytes of streptozotocin-treated (STZ) rats in response to insulin. The insulin-induced shift in titin isoform expression was blocked by a PI3K inhibitor, suggesting that insulin regulates titin isoform composition in the heart through activation of the PI3K/Protein Kinase B (PKB) signaling pathway. Additionally, enhanced titin phosphorylation was observed in insulin-treated cardiomyocytes.

The pathogenesis of type 2 diabetes mellitus unfolds over several years, with progression typically occurring silently and asymptomatically in the background. Insulin resistance and the resulting hyperinsulinemia precede the rise in fasting blood glucose levels by several years [22,23].

Multiple mechanisms have been implicated in the development of diabetic cardiomyopathy [24]. Numerous studies have addressed the role of cardiovascular autonomic neuropathy in the pathophysiology of diabetic cardiomyopathy. However, all publications note the complexity of investigating this issue. The disease does not occur independently in diabetes, and several comorbid conditions unrelated to but often coexisting with diabetes also contribute to its development [25]. In the early stages of cardiovascular autonomic neuropathy, a decrease in parasympathetic tone leads to tachycardia, which may temporarily improve myocardial contractility and relaxation. However, this is later followed by progressive deterioration of left ventricular function [26]. According to echocardiographic findings, the early course of the disease is characterized by impaired left ventricular diastolic function, while systolic function remains preserved [27]. However, similarly to systolic heart failure, this condition is associated with increased mortality [28]. Based on the data from the DCCT/EDIC study, Pop-Busui et al. [29] reported that in individuals with long-standing type 1 diabetes, the presence of cardiovascular autonomic neuropathy (CAN) is associated with left ventricular remodeling and hypertrophy. Similar studies have been conducted in type 2 diabetes as well, where echocardiographic assessments demonstrated that left ventricular diastolic diameter is increased in the presence of CAN [30]. Further studies are needed to clarify the independent impact of cardiovascular autonomic neuropathy on the development of diabetic cardiomyopathy.

The two intronic SNPs in the titin gene (rs2291313 and rs4471922) might indirectly influence the diastolic dysfunction observed in the presence of diabetic cardiomyopathy.

### 3.2. rs6086563 SNP of Phospholipase C-Beta 1 Gene

rs6086563 is an intronic SNP in the phospholipase C-beta 1 (*PLCβ1*) gene. The phospholipase C-beta protein catalyzes the production of inositol-1,4,5-trisphosphate (IP3) and diacylglycerol (DAG) from phosphatidylinositol-4,5-bisphosphate (PIP2), which is a key step in various intracellular signaling pathways, including the intracellular transport of neurotransmitters and hormones that modulate the development and functionality of the central nervous system [31]. PLCβ1 is most prominently expressed in brain regions responsible for higher-order central nervous system functions, such as the frontal cortex, hippocampus, and amygdala. It may play a key role in emotional and cognitive behavior. Based on these findings, impaired phosphoinositide signaling may contribute to the pathology of human psychiatric disorders [32]. Homozygous or compound heterozygous loss-of-function mutations in phospholipase C-β1 have been reported in three children diagnosed with early-onset epileptic encephalopathy [33].

Disruptions in inositol metabolism have been associated with insulin resistance and the microvascular complications of diabetes [34]. In diabetic patients, inositol depletion has been observed [35,36]. Concurrent with myo-inositol depletion, reductions in Na^+^-K^+^-ATPase activity and nerve conduction velocity have been reported, accompanied by axonal atrophy, paranodal swelling, and paranodal demyelination [37].

The rs6086563 SNP identified in the phospholipase C-beta 1 gene may affect myo-inositol depletion, thereby increasing the risk of developing microvascular complications such as neuropathy.

### 3.3. rs4241602 SNP of Cyclin I Gene

rs4241602 is also an intronic SNP in the cyclin I gene. Cyclin I is an atypical cyclin that is most abundantly expressed in postmitotic cells. The cyclin I–Cdk5 complex forms a critical antiapoptotic factor in terminally differentiated cells, playing a role in the modulation of the levels of the antiapoptotic pro-survival proteins B-cell leukemia/lymphoma 2 protein (Bcl-2) and B-cell lymphoma-extra Large (Bcl-XL) through, mitogen-activated protein kinase (MAPK) signaling [38]. Cyclin-dependent kinase 5 (CDK5) affects neuronal apoptosis and plays a role in the development of Alzheimer’s disease, amyotrophic lateral sclerosis, and ischemic stroke [39]. Under normal conditions, CDK5 remains in an inactive state. Upon binding to p35, it becomes activated and subsequently phosphorylates multiple substrates, thereby influencing neuronal development, as well as axonal and dendritic growth. In response to pathological neuronal stimulation, intracellular calcium levels increase, leading to the conversion of p35 into p25. The p25–CDK5 complex results in a hyperactivated state of CDK5, causing widespread intracellular hyperphosphorylation and contributing to the development of neurological diseases [40]. CDK5 influences pain perception in trigeminal ganglion neurons. Increased CDK5 activity in neurons enhances calcium influx, raising the pain sensitivity threshold and increasing the activity of polymodal nociceptors. This process contributes to the development of allodynia, a common feature of neuropathic pain [41].

The use of CDK5 inhibitors in both in vitro and in vivo studies has demonstrated neuroprotective effects, suggesting that CDK5 may serve as a potential therapeutic target for neurological diseases in the future [40].

Our results suggest a potential role for the intronic rs4241602 SNP in the development of allodynia.

### 3.4. rs2396295 and rs892204 SNPs of Cell Division Cycle 34 Gene

The rs2396295 and rs892204 intronic SNPs are located in the cell division cycle 34 (*CD34*) gene. The CD34 gene encodes a transmembrane phosphoglycoprotein that is primarily recognized as a marker of hematopoietic stem and progenitor cells. CD34 is a highly glycosylated cell-surface antigen with a molecular weight of approximately 115 kDa [42,43]. It plays a role in cell adhesion, signal transduction, and the regulation of differentiation and proliferation [44]. CD34 is also expressed on endothelial progenitor cells, fibrocytes, and muscle satellite cells, as well as on interstitial cells and epithelial progenitor cells. It is also found on tumor cells, suggesting that it may play a role in the development of cancer [42,44].

The level of circulating CD34-positive endothelial progenitor cells (EPCs) may provide clinical information about the extent of atherosclerosis and future cardiovascular risk [45]. The relationship between endothelial progenitor cells and diabetes may be mediated by oxidative stress and vascular damage [46]. In diabetes, the number of EPCs is reduced, which can be attributed to several factors, including impaired mobilization from the bone marrow, decreased proliferation, and increased apoptosis [47]. Hyperglycemia reduces antioxidant defense mechanisms, promotes excessive production of reactive oxygen species (ROS), and creates an unfavorable vascular environment that impairs the function of EPCs. All of this contributes to the vascular complications and reduced regenerative capacity observed in patients with diabetes [48].

In their study, Gadau et al. demonstrated that benfotiamine therapy counteracts diabetes-induced endothelial progenitor cell deficiency in diabetic mice [49]. Furthermore, Marchetti et al. established that hyperglycemia-induced EPC damage can be reversed through the administration of benfotiamine via the modulation of PKB/Forkhead box protein O1 (FoxO1) activity [50].

Therefore, the rs2396295 and rs892204 SNPs located in the *CD34* gene may affect endothelial progenitor cell function and vascular repair, particularly in diabetes.

### 3.5. rs6682221 SNP of Anti-Proliferation Factor 2 Gene

rs6682221 is a sequence variant located within the 2KB region at the 5′ end of the anti-proliferation factor 2 (*BTG2*) gene (upstream variant). The BTG anti-proliferation factor 2 gene plays a key role in cell proliferation, apoptosis, and cell growth and may also function as a tumor suppressor. BTG2 and protein arginine methyltransferase 1 (PRMT1) regulate neuronal growth through arginine methylation [51]. In BTG2-deficient mice, an accumulation of immature neurons and impaired contextual memory have been observed [52]. BTG2 can bind to the promoter of inhibitor of DNA binding 3 (ID3) and inhibit its expression [52]. In a study by Zelin Chen et al., it was shown that during wound healing, ID3 expression increases, leading to the activation of dermal fibroblasts and the promotion of neuronal regeneration [53].

Based on our results, the rs6682221 SNP located in the 2KB region at the 5′ end of the BTG anti-proliferation factor 2 gene might play a role in the mechanism of neuronal regeneration.

Among the SNPs we identified, six are intronic variants, which means that they are located in regions of the genes and are not transcribed. However, the rs6682221 SNP, which has been identified in B-cell translocation gene 2, is a sequence variant located within 1000 base pairs of the transcription start site at the 5′ end of the gene. The rs922984 variant in the titin gene is a benign missense variant, so it results in the incorporation of a different amino acid at a specific position, without altering the function of the protein. Nevertheless, these SNPs may be part of higher-level regulatory mechanisms that could indirectly influence pathophysiological processes involved in the development of neuropathy.

Our study represents a significant innovation, as it is the first to systematically map the genetic background of diabetic neuropathy in a population cohort. The SNP patterns and potential genetic risk scores identified in our research are not merely exploratory; they lay the groundwork for the stratification of patients based on genetic predisposition. Obviously, factors other than genetic ones may also contribute to the development of neuropathy, as it is the case in other diseases; however, genetic variants that are associated with this condition, accompanied by such high fold change and significance as in our study, cannot be ignored.

This genetic profiling could play a critical role in precision medicine by supporting the classification of disease subtypes and helping to identify individuals at high risk who may benefit from early intervention—even before clinical symptoms of neuropathy appear. This is particularly relevant for a complication like diabetic neuropathy, which is often diagnosed only at advanced stages, when therapeutic options are already limited.

We fully acknowledge the importance of population-specific genetic variation. Our study highlights the need to consider such differences in future therapeutic developments. The genetic associations identified in this work could guide the development of risk profiles tailored to different ethnic groups, thereby contributing to more personalized and effective treatment strategies.

In summary, our study goes beyond mere detection. It introduces a novel genetic framework that may support earlier diagnosis, prognosis, and therapeutic decision-making, potentially improving outcomes for patients at risk of diabetic neuropathy.

This study has certain limitations—most notably, the relatively small sample size. Despite this, the findings yielded statistically significant results. A follow-up study is currently underway to validate and replicate our findings. A minor disparity was observed in the mean age between the two cohorts. Nevertheless, the neuropathy group was followed for an extended period (mean: 10.3 ± 6.2 years), and the non-neuropathy group for an even longer duration (mean: 13.2 ± 7.5 years). This prolonged follow-up minimizes the likelihood that such an age difference introduced any meaningful bias. Although epigenetic influences could potentially confound the outcomes, the close clinical similarity between the two groups suggests this is unlikely. It should also be noted that the study exclusively included individuals with type 2 diabetes, limiting the generalizability of the results to those with type 1 diabetes.

Among the strengths of this research is its novelty; to the best of our knowledge, this is the first study to apply a comprehensive whole-exome sequencing strategy to explore genetic contributors to diabetic neuropathy in patients with type 2 diabetes.

## 4. Materials and Methods

### 4.1. Patient Selection

A total of 24 patients with diabetic neuropathy (male/female: 17/7; mean age: 66.5 ± 9.27 years; BMI: 31.5 ± 5.0 kg/m^2^; average diabetes duration: 10.3 ± 6.2 years; HbA1c: 7.49 ± 1.09%) and 24 patients without neuropathy (male/female: 13/11; mean age: 56.2 ± 10.8 years; BMI: 30.0 ± 5.2 kg/m^2^; average diabetes duration: 13.2 ± 7.5 years; HbA1c: 7.04 ± 1.0%) with type 2 diabetes were included in our study.

The study protocol was approved by the local ethics committee (number 37596-8/2018/EÜIG), and all participants provided written informed consent after receiving appropriate information.

### 4.2. Neurological Assessment

All study participants underwent a comprehensive neurological evaluation to exclude carpal tunnel syndrome and to identify any signs or symptoms indicative of neurological impairment.

Patients taking medications that could affect the results of parasympathetic autonomic reflex tests based on heart-rate variability (primarily beta-blockers or non-dihydropyridine calcium channel blockers) were asked to withhold these medications for 24 h prior to the examinations.

The clinical examination focused particularly on detecting muscle atrophy and skin changes. Sensory nerve function was assessed using a Neurometer^®^ CPT instrument (Neurotron Inc., Baltimore, MD, USA). Current perception thresholds (CPTs) were measured at the median and peroneal nerves using three stimulus frequencies: 2000 Hz, 250 Hz, and 5 Hz. These frequencies selectively evaluate the function of large myelinated (Aβ), small myelinated (Aδ), and unmyelinated (C) sensory nerve fibers [54,55,56]. Reference values for current perception threshold measurements at all three frequencies for both the peroneal and median nerves were established according to Evans et al. [57]. Thermal detection thresholds for cold and heat were assessed using a Thermal Sensory Analyzer II (TSA-II; Medoc Ltd., Ramat Yishai, Israel). The vibration perception threshold (VPT) was measured with a Vibratory Sensory Analyzer (VSA-3000) on the same device platform. Sensory nerve dysfunction was defined as the presence of at least two abnormal sensory measurements in either the upper or lower limbs across any of the tested frequencies. The severity of neuropathic symptoms was quantified using the Neuropathy Total Symptom Score-6 (NTSS-6), a validated questionnaire that evaluates the frequency and intensity of six common sensory symptoms reported by individuals with diabetic peripheral neuropathy (DPN): sharp, shooting, or lancinating pain; aching pain and/or tightness; allodynia and/or hyperalgesia; prickling and/or tingling sensations; numbness and/or insensitivity; and burning sensations [58].

Cardiovascular autonomic neuropathy (CAN) was assessed using five standard cardiovascular reflex tests. Parasympathetic function was evaluated by measuring heart-rate responses to deep breathing (beat-to-beat variation), active standing (30:15 ratio), and the Valsalva maneuver (Valsalva ratio). Sympathetic function was assessed through blood pressure responses to active standing and sustained handgrip. It should be noted that, according to the Toronto Consensus Panel on Diabetic Neuropathy, the blood pressure response to sustained handgrip is no longer recommended as a clinical diagnostic test and is considered investigational only [59]. All cardiovascular reflex tests were conducted using a Cardiosys H-01 12-lead portable electrocardiograph (ECG) system. A diagnosis of cardiovascular autonomic neuropathy (CAN) was established if at least one cardiovascular reflex test yielded an abnormal result [56,60]. The normative reference values for Neurometer assessments and cardiovascular reflex tests are presented in Table 3 and Table 4, respectively.

### 4.3. Genetic Analysis

#### 4.3.1. DNA Isolation

Genomic DNA was extracted from peripheral blood samples using a HighPure DNA Isolation Kit (Roche, Rotkreuz, Switzerland), following the manufacturer’s protocol. DNA concentration was measured with a Qubit dsDNA High Sensitivity Assay Kit (Thermo Fisher Scientific, Waltham, MA, USA).

#### 4.3.2. Whole-Exome Sequencing (WES)

Whole-exome sequencing was conducted at the Molecular Biology Laboratory, Department of Internal Medicine and Oncology, Semmelweis University, which is equipped with the necessary expertise and infrastructure to support the procedure.

Whole-exome sequencing was performed using the Thermo Ion GeneStudio S5 platform, targeting coding regions of approximately 32,000 genes, corresponding to around 50 million base pairs per patient.

Exome library preparation was performed using an Ion Torrent AmpliSeq RDY Exome Kit (Thermo Fisher Scientific, Waltham, MA, USA), which was used to amplify the target regions of the exome. Genomic DNA concentration was assessed using a Qubit DNA HS or BR Assay Kit (Life Technologies, Carlsbad, CA, USA), and 100 ng of DNA per sample was used for library construction. Ion Xpress barcode adapters were ligated to uniquely label each sample. Following amplification, libraries were purified with AMPure XP beads and quantified using a Qubit 2.0 fluorometer. Final libraries were diluted to approximately 100 pM. Three barcoded libraries were pooled and loaded onto a single Ion 540 chip. Sequencing was performed on the Ion GeneStudio S5 platform (Thermo Fisher Scientific).

Following sequencing, AmpliSeq Exome reads were aligned to the human reference genome (hg38). Coverage analysis and initial data processing were carried out using Ion Torrent Suite Software version 5.12. The resulting Binary Alignment Map (BAM) files were then uploaded to the cloud-based Ion Reporter Software version 5.12, where variant calling and raw data analysis were performed using the Ion Reporter™ AmpliSeq Exome workflow.

Variant annotation was performed using a series of bioinformatic filters. These included single-nucleotide variant (SNV) filtering; assessment of synonymous and missense variant effects; application of functional prediction scores such as SIFT, PolyPhen, and Grantham; restriction of homopolymer length to ≤6 bases; and evaluation of homozygosity, in addition to requiring a minimum allele read count of 100, an allele ratio of 1.0, and a minor allele frequency (MAF) threshold of ≤0.5.

### 4.4. Bioinformatic and Statistical Methods

Variant annotation was carried out using ANNOVAR (v03dec2019), incorporating data from the dbSNP, ClinVar, gnomAD, and OMIM databases. Sequencing reads were visualized using the Integrative Genomics Viewer (IGV), and duplicate reads were marked with Picard tools. Single-nucleotide polymorphism (SNP) calling was performed on Variant Call Format (VCF) files generated using GATK. These VCF files were merged using BCFtools, and the resulting variants were further annotated with SnpSift [61].

Variant annotation was performed using the dbSNP reference database (hg38, build 151) obtained from the NCBI dbSNP repository.

Quality control of the raw VCF files was conducted using PLINK v1.9 [62]. Predicted sample sex based on SNP data was compared to the reported phenotypic sex of the individuals to identify discrepancies. SNP filtering was performed using thresholds for the missingness rate (>0.05), minor allele frequency (MAF < 0.01), and Hardy–Weinberg equilibrium (HWE) *p*-value (<1 × 10^−10^). These thresholds were applied in accordance with the PLINK v1.9 documentation recommendations [63].

Association testing was performed in the R v4.0.3 environment using the GENESIS Bioconductor package to fit logistic regression models [64,65]. To exclude potential confounding effects and account for associations identified during quality control analysis, model estimates were adjusted for age, sex, and relatedness. For the latter, the genetic relatedness matrix (GRM) was generated using the SNPRelate package [66].

The Quantile–Quantile (QQ) plot was constructed using the qqman R package, while the Manhattan plot was created using ggplot2 [67,68]. The newer, most significant SNPs were ranked based on the *p*-values from the logistic regression analysis.

All statistical analyses were performed using R v4.0.3. Continuous variables are expressed as standard deviations and means. Statistical significance was assessed using the Mann–Whitney test. Categorical variables are presented as frequencies, and differences between groups were evaluated using Fisher’s exact test. Statistical significance was determined for test results with a *p*-value less than 0.05.

## 5. Conclusions

Our study provides further insights into the associations between genetic variants and both sensory nerve fiber functions and cardiovascular autonomic neuropathy in diabetes. Our findings suggest that additional specific genetic factors might play a significant role in the pathophysiology of these conditions. Identifying genetic predisposition factors involved in the development of neuropathy is an important step toward personalized medicine. This knowledge offers the possibility of identifying individuals at higher risk of developing neuropathy, even before the onset of symptoms. Optimization of patient management—including metabolic control, more intensive antihypertensive and antilipidemic therapy, smoking cessation, stricter weight control, and the earlier application of available neuropathy treatments—may improve quality of life and increase life expectancy in the long term. Early detection and modern treatment of diabetic foot ulcers can prevent lower-limb amputations. In the future, these genetic variants might be useful not only in risk assessment but also in the development of new pharmacological treatments, as they can help to better understand the underlying processes involved in the development of neuropathy. In summary, despite certain limitations, such as a relatively small sample size and the inclusion of only type 2 diabetic patients, our study provides novel insights into the potential genetic underpinnings of diabetic neuropathy, supported by significant findings. Importantly, this represents the first application of whole-exome sequencing in this context. To strengthen and validate these findings, a follow-up study involving a larger patient cohort is currently in progress.

## Figures and Tables

**Table 1:** Demographic and clinical characteristics of the two study groups.

	T2DM with Neuropathy (n = 24)		T2DM Without Neuropathy (n = 24)		
	Average	±SD	Average	±SD	*p* Value
Age (years)	66.5	9.27	56.2	10.8	0.0012
Body mass (kg)	93.8	15.8	86.8	17.4	0.1009
Body height (cm)	172.5	9.9	170.0	10.5	0.4030
BMI (kg/m^2^)	31.5	5.00	30.0	5.2	0.1670
Systolic blood pressure (Hgmm)	137.7	15.7	134.0	12.2	0.3842
Diastolic blood pressure (Hgmm)	71.3	7.1	75.0	9.1	0.1718
Duration of diabetes (years)	10.3	6.2	13.2	7.5	0.1322
Sex (male/female)	17/7		13/11		
Fasting blood sugar (mmol/L)	8.92	2.81	8.97	3.18	0.9912
HbA1c (%)	7.49	1.09	7.04	1.00	0.1376
Cholesterol (mmol/L)	4.80	0.88	5.05	1.23	0.5596
LDL cholesterol (mmol/L)	2.91	0.82	3.2	0.94	0.4480
HDL cholesterol (mmol/L)	1.26	0.36	1.17	0.29	0.6441
Triglyceride (mmol/L)	1.85	0.88	2.53	1.73	0.3065

Data are reported as mean ± standard deviation (SD) or median [interquartile range, IQR]. Between-group differences were assessed using the χ^2^ test.

**Table 2:** Results of whole-exome sequencing. Summary of identified genetic variants, including chromosomal location, variant position, reference allele frequencies, and associated odds ratios (ORs) for neuropathy risk.

Variant ID	Reference/ Alternative Allele	Position	Gene	Reference Allele Frequency (MAF) of European Population *	Logistic Regression Estimate (β)	Logistic Regression Estimate (β) Standard Error	OR for Reference Allele	*p* Value
rs922984	T/C	chr2:178751160 (GRCh38.p14)	*TTN*	0.070	3.248	0.989	26.69	0.001
rs2291313	T/C	chr2:178767983 (GRCh38.p14)	*TTN*	0.202	2.304	0.738	22.65	0.002
rs4471922	G/T	chr2:178768571 (GRCh38.p14)	*TTN*	0.205	2.304	0.738	22.65	0.002
rs6086563	C/G	chr20:8722498 (GRCh38.p14)	*PLCB1*	0.243	2.787	0.855	25.99	0.001
rs4241602	A/G	chr4:77066198 (GRCh38.p14)	*CCNI*	0.081	4.020	1.264	24.01	0.001
rs2396295	A/G	chr19:536437 (GRCh38.p14)	*CDC34*	0.088	3.213	0.996	25.16	0.001
rs892204	G/A	chr19:536900 (GRCh38.p14)	*CDC34*	0.081	3.213	0.996	25.16	0.001
rs6682221	C/A	chr1:203305408 (GRCh38.p14)	*BTG2*	0.099	−2.761	0.893	0.045	0.002

The table presents the chromosomal location and position of each genetic variant, along with minor allele frequencies and the associated risk of developing neuropathy, as indicated by the odds ratio (OR). * Minor allele frequencies are based on the European population data from the ALFA (Allele Frequency Aggregator) project [14].

**Table 3:** Internationally accepted normative values for current perception threshold (CPT) measurements using a Neurometer device (note: 100 CPT units = 1 mA) [57].

Current Perception Threshold (Frequency)	Nervus Medianus (Normal Range in mm/s)	Nervus Peroneus (Normal Range in mm/s)
2000 Hz	120–398	179–523
250 Hz	22–189	44–208
5 Hz	16–101	18–170

**Table 4:** Normal values for cardiovascular reflex testing [60].

Method	Tested Parameter	Normal Value	Borderline Value	Abnormal Value
Tests for the investigation of parasympathetic functions				
Deep breathing test	Beat-to-beat variation (beats/min)	≥15	11–14	≤10
Valsalva maneuver	Valsalva ratio	≥1.21	1.11–1.2	≤1.1
Heart-rate response to standing	30/15 ratio	≥1.04	1.01–1.03	≤1.0
Tests for the investigation of sympathetic functions				
Blood pressure (BP) response to standing	Reduction in systolic BP (mmHg)	≤10	11–29	≥30
Handgrip test	Increase in diastolic BP (mmHg)	≥16	11–15	≤10

## Data Availability

Data is contained within the article.
